# *Bacteroides ovatus* alleviates dysbiotic microbiota-induced intestinal graft-versus-host disease

**DOI:** 10.21203/rs.3.rs-2460097/v1

**Published:** 2023-01-31

**Authors:** Eiko Hayase, Tomo Hayase, Akash Mukherjee, Stuart C. Stinson, Mohamed A. Jamal, Miriam R. Ortega, Christopher A. Sanchez, Saira S. Ahmed, Jennifer L. Karmouch, Chia-Chi Chang, Ivonne I. Flores, Lauren K. McDaniel, Alexandria N. Brown, Rawan K. El-Himri, Valerie A. Chapa, Lin Tan, Bao Q. Tran, Dung Pham, Taylor M. Halsey, Yimei Jin, Wen-Bin Tsai, Rishika Prasad, Israel K. Glover, Nadim J. Ajami, Jennifer A. Wargo, Samuel Shelburne, Pablo C. Okhuysen, Chen Liu, Stephanie W. Fowler, Margaret E. Conner, Christine B. Peterson, Gabriela Rondon, Jeffrey J. Molldrem, Richard E. Champlin, Elizabeth J. Shpall, Philip L. Lorenzi, Rohtesh S. Mehta, Eric C. Martens, Amin M. Alousi, Robert R. Jenq

**Affiliations:** 1 Department of Genomic Medicine, The University of Texas MD Anderson Cancer Center, Houston, Texas 77054, USA; 2 Department of Stem Cell Transplantation and Cellular Therapy, The University of Texas MD Anderson Cancer Center, Houston, Texas 77030, USA; 3 Metabolomics Core Facility, Department of Bioinformatics and Computational Biology, The University of Texas MD Anderson Cancer Center, Houston, Texas 77230, USA; 4 Department of Infectious Diseases, Infection Control and Employee Health, The University of Texas MD Anderson Cancer Center, Houston, Texas 77030, USA; 5 Department of Pathology, Yale School of Medicine, New Haven, Connecticut 06520, USA; 6 Department of Molecular Virology and Microbiology, Baylor College of Medicine, Houston, TX 77030, USA.; 7 Center for Comparative Medicine, Baylor College of Medicine, Houston, TX 77030, USA.; 8 Department of Biostatistics, The University of Texas MD Anderson Cancer Center, Houston, Texas 77030, USA; 9 Department of Microbiology & Immunology, University of Michigan Medical School, Ann Arbor, Michigan 48109, USA; 10 CPRIT Scholar in Cancer Research, Houston, Texas, USA

**Keywords:** *Bacteroides ovatus*, *Bacteroides thetaiotaomicron*, allogeneic hematopoietic stem cell transplantation, graft-versus-host disease, carbapenem, intestinal microbiome, mucus layer, xylose, polysaccharides, polysaccharide utilization loci

## Abstract

Acute gastrointestinal intestinal GVHD (aGI-GVHD) is a serious complication of allogeneic hematopoietic stem cell transplantation, and the intestinal microbiota is known to impact on its severity. However, an association between treatment response of aGI-GVHD and the intestinal microbiota has not been well-studied. In a cohort of patients with aGI-GVHD (n=37), we found that non-response to standard therapy with corticosteroids was associated with prior treatment with carbapenem antibiotics and loss of *Bacteroides ovatus* from the microbiome. In a mouse model of carbapenem-aggravated GVHD, introducing *Bacteroides ovatus* reduced severity of GVHD and improved survival. *Bacteroides ovatus* reduced degradation of colonic mucus by another intestinal commensal, *Bacteroides thetaiotaomicron*, via its ability to metabolize dietary polysaccharides into monosaccharides, which then inhibit mucus degradation by *Bacteroides thetaiotaomicron* and reduce GVHD-related mortality.

## Introduction

Graft-versus-host disease (GVHD) is a common complication in patients undergoing allogeneic hematopoietic stem cell transplantation (allo-HSCT) and occurs when donor T cells recognize the patient’s tissues as foreign. The intestine is often targeted, and severe acute gastrointestinal GVHD (aGI-GVHD) tends to have a poor prognosis. Approximately half of aGI-GVHD cases do not respond to first-line steroid therapy, leading to a high risk for severe complications and reduced overall survival^1,2^. Novel immune suppression strategies to treat steroid-refractory GVHD have been established, including Janus kinase 1/2 (JAK1/2) inhibitors, with demonstrated clinical efficacy, though not all patients will respond^3,4^.

The intestinal microbiota is an important modulator of the host immune system^5,6^ and modulates the pathophysiology of GVHD^7^. Patients undergoing allo-HSCT are at high risk for perturbations in the intestinal microbiota resulting from a number of factors; chief amongst them exposure to antibiotics for prevention and treatment of bacterial infections post-transplant. Broad-spectrum antibiotics such as carbapenems have been reported to increase the incidence of aGI-GVHD^8–11^. Recently, fecal microbiota transplantation has been shown to result in improvement in GVHD in steroid-refractory patients^12–14^, suggesting that the intestinal microbiota can modulate aGI-GVHD treatment responsiveness. It remains unclear, however, how intestinal microbial composition can modulate treatment response of aGI-GVHD.

In this study, we aimed to evaluate for an impact of intestinal microbiota at the onset of aGI-GVHD on GVHD severity. Our retrospective analysis of 37 aGI-GVHD patients showed that steroid-refractory GVHD was significantly associated with higher clinical stages and histological grades of aGI-GVHD at the onset of aGI-GVHD and prior treatment with carbapenem-class antibiotics such as meropenem before onset of aGI-GVHD was significantly associated with steroid-refractory GVHD. An examination of the intestinal microbiome collected from aGI-GVHD patients at the onset of aGI-GVHD revealed that steroid-refractory patients showed greater dysbiosis than responsive patients and high abundances of *Bacteroides ovatus* were significantly associated with improved response to steroid therapy in aGI-GVHD patients.

We recently found that in a murine GVHD model, treatment with meropenem, a commonly used carbapenem in allo-HSCT patients, expanded a mucus-degrading bacterial species, *Bacteroides thetaiotaomicron* (*B. theta*), and aggravated colonic GVHD^15^. Using this model, we evaluated for the impact of *B. ovatus* on GVHD severity in mice with meropenem-aggravated colonic GVHD. Consistent with the clinical findings, we found that introduction of *B. ovatus* improved survival of mice with meropenem-aggravated colonic GVHD. *B. ovatus* also inhibited the expansion and mucus-degrading functionality of *B. theta*. Meropenem altered not only the microbiome composition but also the intestinal environment, including the levels of carbohydrates, increasing mucus-degrading functionality by *B. theta*. Thus, altered functions of intestinal microbes due to changes of metabolic substrates in the colonic lumen can strongly modulate GVHD severity. *B. ovatus* has been reported to have a different spectrum of polysaccharide-degrading functions compared to that of *B. theta*^16^. Importantly, we confirmed via in vitro assay that *B. ovatus* unlike *B. theta* did not show mucus-degrading functionality. Indeed, medium containing xylose-comprising polysaccharides and conditioned by *B. ovatus* could suppress the mucus-degrading functionality of *B. theta* in vitro. The ability of *B. ovatus* to degrade xylose-comprising polysaccharides and produce abundant monosaccharides including xylose in the colonic lumen may play a key role in improving the intestinal metabolic environment in allo-HSCT and prevent expansion of *B. theta*, leading to favorable outcomes of aGI-GVHD.

## Results

### *Bacteroides*-enriched microbiome was associated with favorable treatment response of aGI-GVHD in allo-HSCT patients

To investigate the potential impact of intestinal microbiome composition on aGI-GVHD treatment response, we retrospectively studied patients at MD Anderson Cancer Center who developed aGI-GVHD in the setting of allo-HSCT from 2017 to 2019. A total of 37 patients were diagnosed with aGI-GVHD (Supplementary Table 1): 28 with classic aGI-GVHD and 9 with late-onset aGI-GVHD, by National Institutes of Health consensus criteria^17^. We determined treatment response as previously reported^18^. All patients received initial therapy with methylprednisolone or prednisone at 2 mg/kg/day followed by tapering per institutional guidelines.

**Extended Data Fig. 1.**
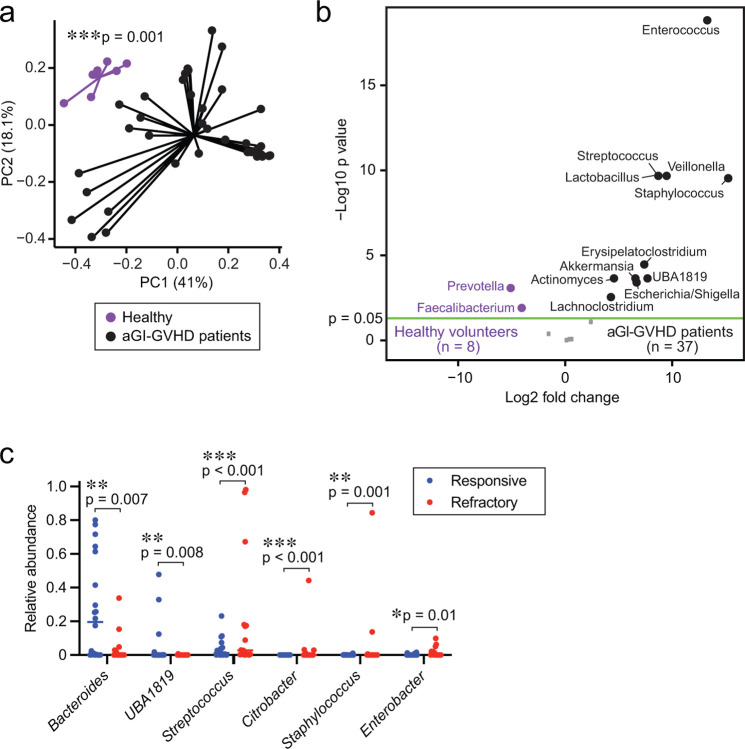
aGI-GVHD patients showed a higher proportion of carbapenem exposure . (**A**) PCoA of fecal samples collected from healthy volunteers or aGI-GVHD patients. (**B**) Volcano plot of differentially abundant genera analyzed by 16S rRNA gene sequencing of fecal samples compared between healthy volunteers and aGI-GVHD patients. (**C**) Relative abundance of genera that were significantly different between steroid-responsive and -refractory aGI-GVHD.

An examination of the microbiome composition of the stool samples using 16S rRNA gene sequencing revealed that our aGI-GVHD cohort showed a significantly distinct intestinal microbiome at the onset of aGI-GVHD from that of healthy volunteers, visualized with principal coordinates analysis (PCoA) and tested using permutational multivariate analysis of variance (PERMANOVA) (Extended Data Fig. 1a). In particular, aGI-GVHD patients showed significantly higher abundance of the genus *Enterococcus* and reductions in the genera *Prevotella* and *Faecalibacterium* (Extended Data Fig. 1b). These results were consistent with previous reports identifying *Escherichia coli* and the genus *Enterococcus* as bacteria that can aggravate GVHD severity^19,20^.

**Fig. 1.**
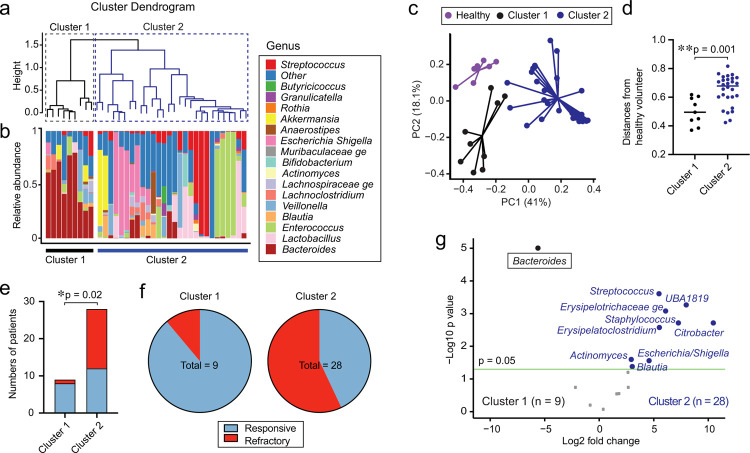
The high abundance of *Bacteroides* was associated with steroid-responsive GVHD. (**a**) Cluster dendrogram analyzed using H-clustering of weighted UniFrac. (**b**) The microbiome composition shown as stacked bar graphs. (**c**) PCoA of fecal samples collected from healthy volunteers or each cluster of aGI-GVHD patients. (**d**) Distances from healthy volunteers in weighted UniFrac. (**e**) Numbers of patients with steroid-responsive and -refractory GVHD. (**f**) Proportions of patients with steroid-responsive and -refractory GVHD. (**g**) Volcano plot of differentially abundant genera between clusters 1 and 2.

We next sought to identify naturally-occurring subsets within aGI-GVHD patients based on differences in microbiome composition. We classified aGI-GVHD patients using hierarchical clustering of weighted UniFrac beta diversity measures and identified 2 distinct groups, with 9 patients in cluster 1 and 28 patients in cluster 2 (Fig. 1a, b). Other than gender (cluster 1 included a significantly higher proportion of male patients; *p* = 0.01; Supplementary Table 2), no clinical transplant characteristics were significantly different between clusters 1 and 2. Interestingly, cluster 1 showed significantly less dysbiosis, as measured by weighted UniFrac from the microbiome of healthy volunteers (Fig. 1c, d). We also found that cluster 1 included a significantly higher proportion of steroid-responsive GVHD patients than cluster 2 (Fig. 1e, f). Performing differential abundance analysis on clusters 1 and 2, we found that cluster 1 was primarily characterized by increased abundance of the genus *Bacteroides* (Fig. 1g). Overall, these findings suggested that the composition of the intestinal microbiome may be associated with treatment response of aGI-GVHD.

We then investigated whether the composition of the intestinal microbiome at the onset of aGI-GVHD was different between patients who would later be steroid-responsive or steroid-refractory. Our aGI-GVHD patient cohort included 20 patients whose aGI-GVHD was steroid-responsive and 17 patients whose aGI-GVHD was steroid-refractory. Other than age (refractory cases were in significantly younger patients; *p* = 0.0002; Supplementary Table 3), no clinical transplant characteristics were significantly different between responsive and refractory cases. The time from allo-HSCT until the onset of aGI-GVHD was a median of 31.5 days (range, 14–367 days) in steroid-responsive patients and 42 days (13–257 days) in steroid-refractory patients.

**Fig. 2.**
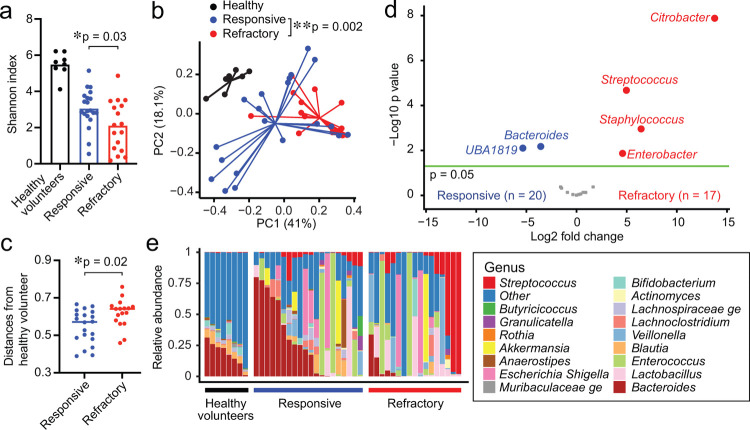
Steroid-refractory aGI-GVHD patients showed significantly dysbiotic intestinal microbiome than steroid-responsive aGI-GVHD patients. (**A-E**) The intestinal microbiome analyzed by 16S rRNA sequencing in patient stool samples collected at presentation with acute intestinal graft-versus-host disease (aGI-GVHD). (**A**) Alpha diversity shown as Shannon index. (**B**) Principal coordinates analysis (PCoA) of fecal samples collected from healthy volunteers or steroid-responsive or steroid-refractory patients. (**C**) Distances from healthy volunteers in weighted UniFrac. (**D**) Volcano plot of differentially abundant genera. (**E**) The composition of the intestinal microbiome.

We found that steroid-responsive patients showed significantly higher microbial alpha diversity than steroid-refractory patients, but that this diversity was still lower than that of healthy volunteers (Fig. 2a). Using PCoA with PERMANOVA testing, we found that the intestinal microbiome was significantly different between steroid-responsive and steroid-refractory patients (Fig. 2b) and that steroid-refractory patients showed greater dysbiosis than responsive patients, as measured by their weighted UniFrac differences from the microbiome of healthy volunteers (Fig. 2b, c). We evaluated the bacterial taxa that were differentially abundant and found that steroid-refractory patients had reductions in the genera *Bacteroides* and *UBA1819* and higher abundances of the genera *Citrobacter, Streptococcus, Staphylococcus*, and *Enterobacter* (Fig. 2d, e and Extended Data Fig. 1c). Overall, these results suggested that alterations of the composition of the intestinal microbiome at clinical presentation of aGI-GVHD were associated with poor response to therapy.

### No prior treatment with carbapenems and higher abundances of *Bacteroides ovatus* were significantly associated with favorable outcomes of aGI-GVHD

**Fig. 3.**
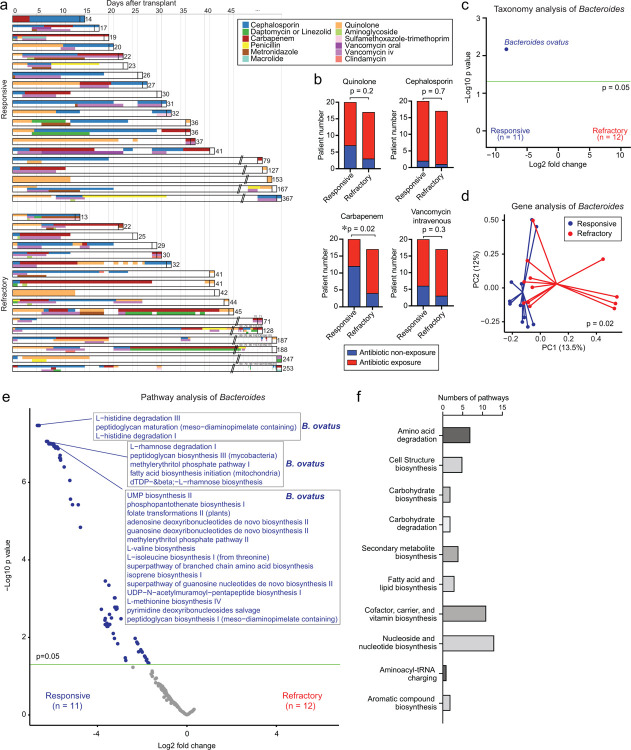
The high abundance of *Bacteroides ovatus* and *B. ovatus*-derived pathways were associated with steroid-responsive GVHD. (**A**) Graphical summary of antibiotics used in individual patients. (**B**) Numbers of patients with antibiotic exposure between hematopoietic stem cell transplant (HSCT) and the onset of GVHD. (**C-F**) Data analyzed by shotgun sequencing of fecal samples collected from aGI-GVHD patients (steroid-responsive; n=11, steroid-refractory; n=12). (**C**) Volcano plot of differentially abundant species between steroid-responsive and - refractory GVHD. (**D**) PCoA of genes in the genus *Bacteroides*. (**E**) Volcano plot of differentially abundant pathways of the genus *Bacteroides*. (**F**) The top 50 subclasses of differentially abundant pathways of the genus *Bacteroides*.

Allo-HSCT patients are often treated with broad-spectrum antibiotics for febrile neutropenia and other infections that arise before as well as after hematopoietic engraftment. These antibiotics, however, can cause bystander damage to intestinal commensals that are critical for maintaining intestinal homeostasis. Indeed, exposure to broad-spectrum antibiotics such as carbapenems has been linked to an increased incidence of aGI-GVHD^8–11^. We examined patient antibiotic treatment histories during the period from allo-HSCT to the onset of aGI-GVHD and looked for associations with steroid response for GVHD (Fig. 3a). Steroid-refractory patients had significantly higher prior treatment with carbapenems but not quinolone, cephalosporin, or intravenous vancomycin (Fig. 3b). Together, these results indicated that antibiotic-mediated microbiome disruption could be an important determinant of response of GVHD to therapy.

To identify specific species of *Bacteroides* potentially associated with steroid response for aGI-GVHD, we performed whole-genome sequencing on only 23 fecal samples which had remained available genomic DNA or stool. In samples from 23 patients, including 11 steroid-responsive patients and 12 steroid-refractory patients, abundances of *B. ovatus* were significantly increased in steroid-responsive patients (Fig. 3c). Analysis of abundances of individual *Bacteroides*-derived genes demonstrated that *Bacteroides* from steroid-responsive patients showed significantly distinct gene contents from that of *Bacteroides* from steroid-refractory patients using PCoA with PERMANOVA testing (Fig. 3d). Evaluation of genetic pathways from *Bacteroides* demonstrated that multiple genetic pathways of *Bacteroides* were significantly associated with steroid-responsive patients but none of them in steroid-refractory patients. Interestingly, the top 50 pathways with significantly increased abundances in steroid-responsive patients, including the pathways related with amino acid degradation and carbohydrate biosynthesis/degradation, belonged to *B. ovatus* (Fig. 3e, f), indicating that *B. ovatus* is particularly associated with steroid-responsive GVHD in patients. In summary, results of 16S rRNA and whole-genome sequencing of patient fecal samples at the onset of aGI-GVHD implicated a potential beneficial effect of *B. ovatus*, which we further examined in a murine GVHD model.

### *B. ovatus* suppressed meropenem-aggravated colonic GVHD in a murine GVHD model

**Extended Data Fig. 2.**
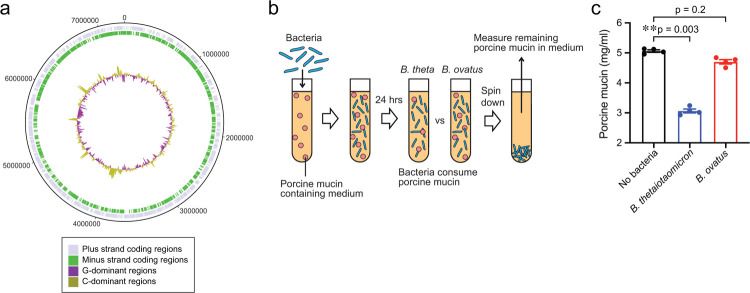
*Bacteroides ovatus* did not show mucus-degrading functionality like *B. theta*. (**A**) Circular plot of open reading frames (ORFs) derived from the complete genome (MDA-HVS BO001). Blue and green bars represent ORFs on the plus strand and the minus strand, respectively. Inner purple-olive ring depicts degree of GC skewing. (**B**) Experimental schema of in vitro bacterial culture assay of *B. theta* (MDA-JAX BT001) or *B. ovatus* (MDA-HVS BO001) in media with porcine gastric mucin-containing medium. (**C**) Relative concentrations of porcine gastric mucin in medium following culture with *B. theta* (MDA-JAX BT001) or *B. ovatus* (MDA-HVS BO001). *B. theta* or *B. ovatus* was first introduced to porcine gastric mucin-containing medium. At 24 hours of culture, levels of mucin glycans in the culture supernatant were determined using a colorimetric assay.

To investigate whether *B. ovatus* influences GVHD outcomes in a murine GVHD model, we isolated *B. ovatus* from the stool of a healthy volunteer and named as MDA-HVS BO001. We assembled the complete genome of MDA-HVS BO001 and confirmed that it was a strain of *B. ovatus*, with 99.4% of the genomic identity of the ATCC strain of *B. ovatus* (ATCC 8483) (Extended Data Fig. 2a). Hereafter, we refer to our isolated *B. ovatus*, MDA-HVS BO001, as *B. ovatus*.

**Fig. 4.**
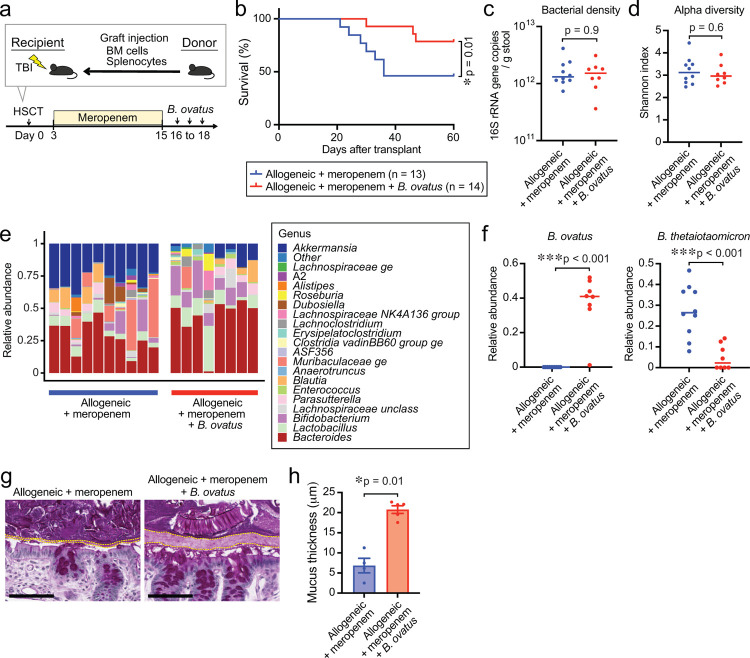
*Bacteroides ovatus* improved GVHD-related mortality in meropenem-aggravated colonic GVHD via suppressing the abundance of *B. theta*. (**A**) Experimental schema of murine GVHD model using meropenem treatment followed by oral gavage of 20 million colony-forming units of *B. ovatus* daily for 3 days. (**B**) Overall survival after allo-HSCT. Data are combined from two independent experiments. (**C**) Bacterial densities of mouse stool samples collected on day 21 after administering meropenem by drinking water. Bacterial densities were measured by 16S rRNA gene qPCR. (**D**) Alpha diversity, measured by the Shannon index, was quantified in fecal samples. (**E**) Bacterial genera composition of fecal samples. (**F**) Relative abundance of *B. ovatus* (left) and *B. theta* (right). (**G**) Periodic acid-Schiff (PAS) staining of histological colon sections collected on day 23. Bar, 100 μm. The areas inside dotted lines indicate the inner dense colonic mucus layer. (**H**) Mucus thickness on day 23. Data are shown from one representative experiment.

As exposure of carbapenems prior to aGI-GVHD onset was significantly associated with the development of steroid-refractory GVHD, we used a meropenem-aggravated GVHD murine model, previously described^15^, to determine the impact of *B. ovatus* on GVHD severity. Briefly, lethally irradiated B6D2F1 (H-2^b/d^) mice were intravenously injected with 5×10^6^ bone marrow cells and 5×10^6^ splenocytes from major histocompatibility complex (MHC)-mismatched B6 (H-2^b^) mice on day 0. Meropenem was administered to the allo-HSCT recipient mice in their drinking water on days 3 to 15 relative to allo-HSCT (Fig. 4a). We previously showed that allo-HSCT mice treated with meropenem demonstrated aggravated colonic GVHD in association with loss of the class Clostridia and expansion of *Bacteroides thetaiotaomicron* (*B. theta*) compared to those untreated with meropenem. *B. theta* is a species of mucus-degrading bacteria that commonly colonizes the intestinal tract of both mice and humans^21^. In this model, expansion of *B. theta* induces thinning of the colonic mucus layer and increases bacterial translocation, leading to aggravated colonic GVHD. To compare mucus-degrading functionality between *B. ovatus* and *B. theta*, we quantified degradation of mucin-derived carbohydrates in vitro using a periodic acid-Schiff (PAS)-based colorimetric assay (Extended Data Fig. 2b). As expected, *B. theta* displayed degradation of mucin-derived carbohydrates, whereas *B. ovatus* did not (Extended Data Fig. 2c), suggesting that *B. ovatus* has less potential to induce mucus-degrading bacteria-related aggravated GVHD.

**Extended Data Fig. 3.**
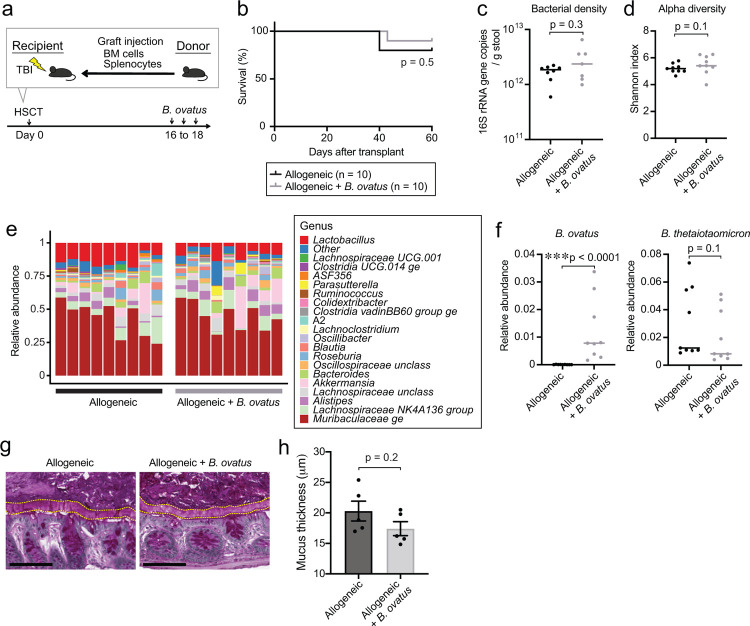
Introduction of *Bacteroides ovatus* did not alter abundance and functionality of *B. theta* in meropenem-untreated allo-HSCT mice. (**A**) Experimental schema of murine GVHD model with oral gavage of 20 million colony-forming units of *B. ovatus* daily from days 16 to 18. (**B**) Overall survival after allo-HSCT. Data are combined from two independent experiments. (**C**) Bacterial densities of mouse stool samples collected on day 21. Bacterial densities were measured by 16S rRNA gene qPCR. (**D**) Alpha diversity, measured by the Shannon index, was quantified in fecal samples. (**E**) Bacterial genera composition of fecal samples. (**F**) Relative abundance of *B. ovatus* (left) and *B. theta* (right). (**B-F**) Combined data from two independent experiments. (**G**) PAS staining of histological colon sections collected on day 23. Bar, 100 μm. The areas inside dotted lines indicate the inner dense colonic mucus layer. (**H**) Mucus thickness on day 23. Data are shown from one representative experiment.

Next, to study the effects of *B. ovatus* on GVHD severity, we orally inoculated 2 × 10^7^ colony-forming units of *B. ovatus* into meropenem-treated allo-HSCT recipient mice from days 16 to 18 and monitored GVHD severity and survival (Fig. 4a). Interestingly, we found that meropenem-treated mice that received *B. ovatus* showed significantly improved survival (Fig. 4b). However, the favorable effects of *B. ovatus* were not seen in allo-HSCT mice not treated with meropenem (Extended Data Fig. 3a, b), suggesting that *B. ovatus* can mitigate the severity of aGI-GVHD only in the context of disrupted microbiota. This finding, together with the finding that expanded *B. theta* after meropenem treatment was associated with aggravated colonic GVHD, indicated that different *Bacteroides* species, which are quite heterogeneous in their metabolic functions, can mediate distinct and even opposing effects on aGI-GVHD^22,23^. We hypothesized that *B. ovatus* mitigate GVHD severity via their functionality for maintaining intestinal homeostasis, which was supported by that *B. ovatus*-derived pathways were significantly associated with steroid response in our whole-genome sequencing analysis.

To elucidate the mechanisms by which *B. ovatus* mitigated meropenem-aggravated colonic GVHD, we examined the abundance and functionality of *B. theta* in both meropenem-treated and untreated allo-HSCT mice with or without introduction of *B. ovatus*. Introduction of *B. ovatus* did not alter bacterial density quantified by 16S rRNA gene quantitative polymerase chain reaction (qPCR), or alpha diversity quantified using the Shannon index, in stool collected on day 21 in meropenem-treated mice or in control allo-HSCT mice untreated with meropenem (Fig. 4c, d and Extended Data Fig. 3c, d). Interestingly, expansion of *B. theta* induced by meropenem was significantly suppressed by administration of *B. ovatus* in meropenem-treated mice (Fig. 4e, f). Consistent with this, the thickness of the colonic mucus layer was significantly increased in meropenem-treated mice that received *B. ovatus* compared to those without *B. ovatus* (Fig. 4g, h). On the other hand, meropenem-untreated allo-HSCT mice showed no significant effects of administration of *B. ovatus* on relative abundance of *B. theta* or colonic mucus layer thickness (Extended Data Fig. 3e–h). These data suggested that *B. ovatus* suppresses the expansion of *B. theta* in mice under certain conditions, such as following meropenem treatment, but otherwise does not impact substantially on *B. theta*.

### Introducing *B. ovatus* suppressed mucus-degrading functionality by *B. theta* in a murine GVHD model

**Extended Data Fig. 4.**
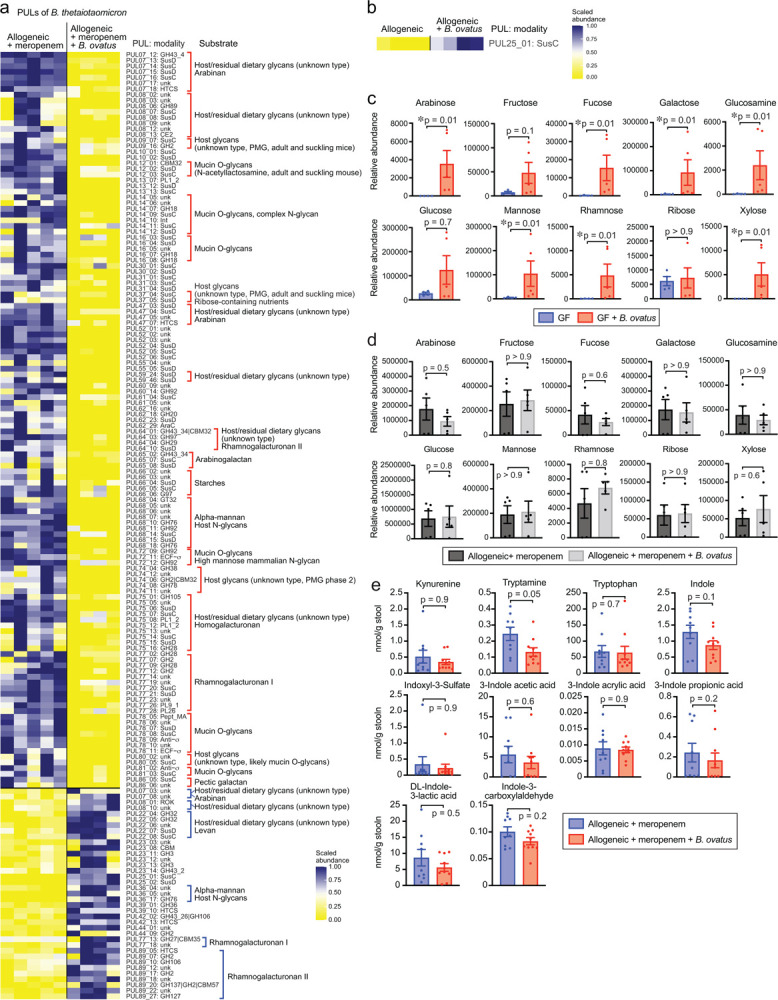
Introduction of *Bacteroides ovatus* increased fecal levels of soluble monosaccharides in mice monocolonized with *B. ovatus*. (**A**) Heatmap showing scaled relative expression levels of polysaccharide utilization loci (PULs) in *B. theta* RNA transcripts sequenced from stool collected from meropenem-treated allo-HSCT mice with or without administration of *B. ovatus* on day 21. Right: PULs and their modularity and substrate names. (**B**) Relative expression levels of PULs in *B. theta* RNA transcripts sequenced from stool collected on day 21 from meropenem-untreated allo-HSCT mice with or without administration of *B. ovatus*. Right: PULs and their modularity. (**C**) Relative abundances of monosaccharides of supernatants from colonic luminal content collected from germ-free (GF) mice with or without administration of *B. ovatus* on day 14 measured by IC-MS. Data are shown from one representative experiment. (**D**) Relative abundances of monosaccharides of supernatants from colonic luminal content collected from meropenem-untreated allo-HSCT mice with or without administration of *B. ovatus* on day 23 measured by ion chromatography-mass spectrometry (IC-MS). Data are shown from one representative experiment. (**E**) Absolute abundances of tryptophan metabolites of supernatants from colonic luminal content collected from meropenem-treated allo-HSCT mice with or without administration of *B. ovatus* on day 23 measured by liquid chromatography coupled with high-resolution mass spectrometry (LC-HRMS). Data are combined from two independent experiments and are shown as means ± SEM.

**Fig. 5.**
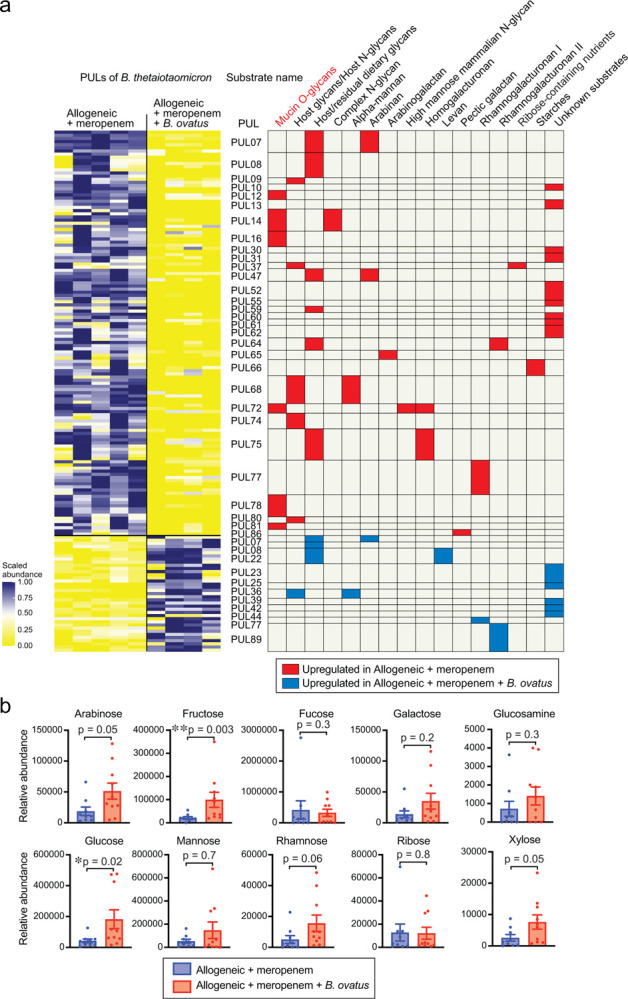
Mucolytic activity of *Bacteroides thetaiotaomicron* is suppressed in meropenem-treated mice by administration of *Bacteroides ovatus*. (**A**) Heatmap showing scaled relative expression levels of polysaccharide utilization loci (PULs) in *B. theta* RNA transcripts sequenced from stool collected from meropenem-treated allo-HSCT mice with or without administration of *B. ovatus* on day 21. Right: Significantly altered PULs and their substrates. (**B**) Relative abundances of monosaccharides of supernatants from colonic luminal content collected from meropenem-treated allo-HSCT mice with or without administration of *B. ovatus* on day 23 measured by ion chromatography-mass spectrometry (IC-MS). Combined data from two independent experiments are shown as means ± SEM.

On the basis of our previous finding that meropenem treatment led to changes in carbohydrate levels and mucus-degrading functionality of *B. theta* in our murine GVHD model^15^, we hypothesized that *B. ovatus* introduction could be impact the carbohydrate environment and *B. theta* gene expression. We began with investigating the effect of *B. ovatus* on *B. theta* gene expression in meropenem-treated allo-HSCT mice, by performing microbial RNA sequencing of stool samples. We examined RNA reads from *B. theta* and annotated these using the polysaccharide utilization loci (PULs) DataBase^24^. We found that administration of *B. ovatus* in meropenem-treated allo-HSCT mice led to downregulation in *B. theta* of many PULs that contribute to degradation of mucin O-glycans, including PULs 12, 16, 78, 81, and parts of 14 (Fig. 5a and Extended Data Fig. 4a). In contrast, in meropenem-untreated mice, administration of *B. ovatus* did not result in downregulation of any of these PULs by *B. theta* which generally showed few transcriptomic changes, which was supported by that *B. theta* in meropenem-untreated mice did not increase the mucus-degrading functionality in our prior study^15^ (Extended Data Fig. 4b). These results suggested that *B. ovatus* not only suppressed expansion of *B. theta* relative abundances, but also produced downregulation of mucus-degrading functionality by *B. theta* in meropenem-treated allo-HSCT mice.

In our previous study of meropenem-aggravated colonic GVHD, we found that mucus-degrading functionality of *B. theta* is repressed by higher concentrations of ambient monosaccharides, including especially xylose^15^. We thus quantified effects of *B. ovatus* on colonic luminal concentrations of monosaccharides using ion chromatography-mass spectrometry (IC-MS). Interestingly, most monosaccharides were markedly increased in meropenem-treated mice that received introduction of *B. ovatus* (Fig. 5b), indicating that *B. ovatus* may be raising concentrations of monosaccharides by helping to degrade dietary-derived polysaccharides. To evaluate if *B. ovatus* was sufficient to elevated monosaccharide concentrations by itself without contributions from other intestinal bacteria, we utilized gnotobiotic mouse models. We measured carbohydrate concentrations of colonic luminal contents collected from previously germ-free (GF) mice two weeks after introduction of *B. ovatus*. We found increased concentrations of many monosaccharides in the colonic lumen of mice monocolonized with *B. ovatus*, while GF mice had very low concentrations of nearly all monosaccharides except ribose (Extended Data Fig. 4c). As expected, monosaccharide concentrations in the colonic lumen of meropenem-untreated mice were not significantly affected by *B. ovatus* introduction (Extended Data Fig. 4d). These results suggested that *B. ovatus* functions in the setting of an injured microbiota to elevate concentrations of monosaccharides in the colonic lumen. It has also been reported that *B. ovatus* can produce indole-3-acetic acid and promote interleukin-22 production from immune cells, leading to decreased colonic inflammation in a murine inflammatory bowel disease model^25^. We did not observe, however, significant changes in concentrations of tryptophan metabolites due to *B. ovatus* in our model (Extended Data Fig. 4e). Thus, our results indicated that *B. ovatus* is effective in elevating concentrations of monosaccharides in the colonic lumen of mice compared to mice with an absent or injured microbiota.

### Degradation of xylose-comprising polysaccharides by *B. ovatus* suppressed mucus-degrading functionality by *B. theta*

**Extended Data Fig. 5.**
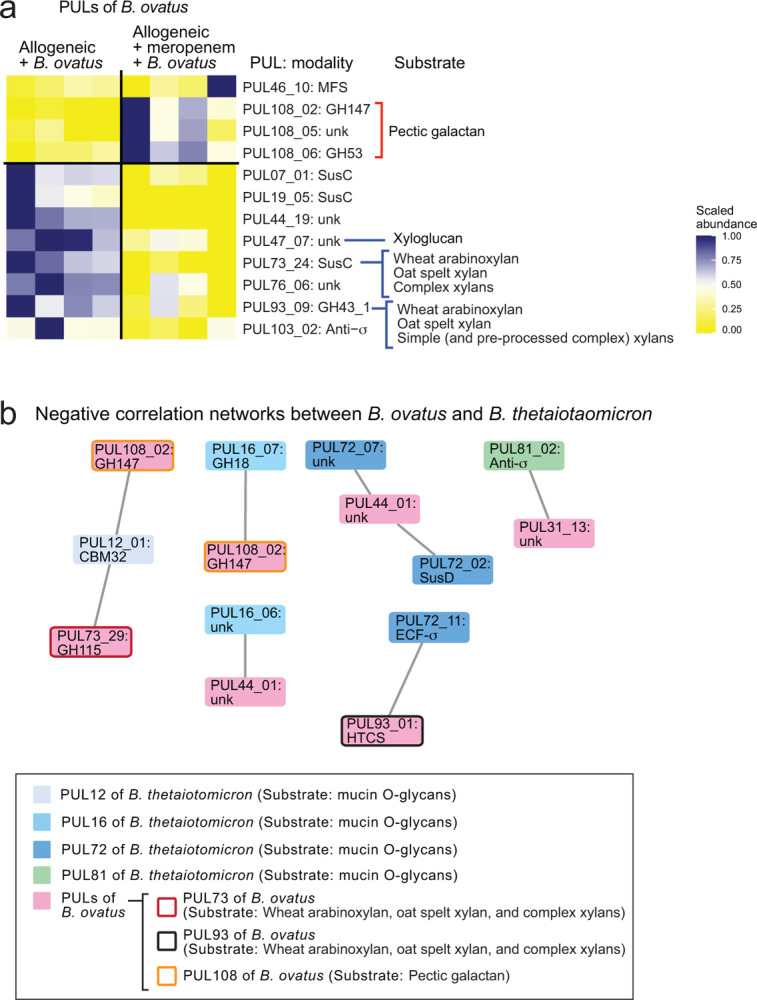
PULs of *Bacteroides ovatus* were significantly altered in meropenem-treated allo-HSCT mice compared to meropenem-untreated mice. (A) Relative expression levels of PULs in *B. ovatus* RNA transcripts sequenced from stool collected from allo-HSCT mice treated or untreated with meropenem on day 28. Right: PULs and their modularity and substrate names. (B) The correlation network analysis of *B. ovatus* RNA transcripts and *B. theta* RNA transcripts sequenced from stool collected on day 21 from meropenem-treated and -untreated allogeneic mice with administration of *B. ovatus*. Only negatively correlated networks are shown.

Interestingly, in contrast to *B. theta*, *B. ovatus* is known to have the ability to degrade xylose-comprising polysaccharides^16,26^. This led us to hypothesize that the ability of *B. ovatus* to degrade xylose-comprising polysaccharides could mediate GVHD. Because *B. ovatus* mediates effects on GVHD severity and on colonic monosaccharides only when introduced to mice following meropenem treatment, we quantified effects of meropenem-pretreatment on gene expression of *B. ovatus* using microbial RNA sequencing of stool samples. We found that *B. ovatus* in meropenem-treated mice upregulated expression of PUL108, which contributes to degradation of pectic galactan, while PULs that perform degradation of xylose-comprising polysaccharides were significantly downregulated compared to in meropenem-untreated allo-HSCT mice (Extended Data Fig. 5a).

**Fig. 6.**
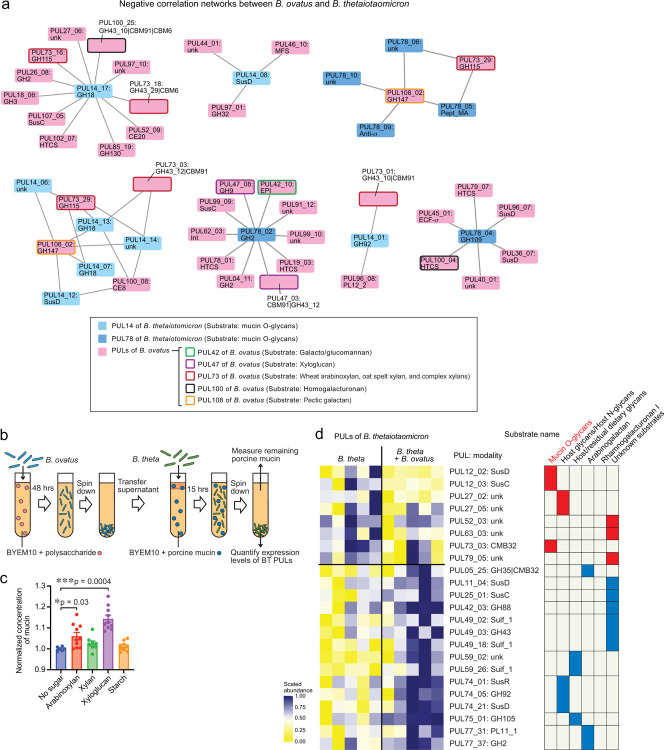
Degradation of xylose-comprising polysaccharides by *Bacteroides ovatus* suppressed mucus-degrading functionality by *Bacteroides thetaiotaomicron*. (**a**) The correlation network analysis of *B. ovatus* RNA transcripts and *B. theta* RNA transcripts sequenced from stool collected on day 21 from meropenem-treated and -untreated allogeneic mice with administration of *B. ovatus*. Only negatively correlated networks are shown. (**b**) Experimental schema of in vitro bacterial culture assay using *B. ovatus* (MDA-HVS BO001) cultured in minimum nutrition medium with each polysaccharide and *B. theta* (MDA-JAX BT001) cultured in BYEM10 with porcine gastric mucin. (**c**) Normalized concentrations of porcine gastric mucin in the culture supernatant were determined using a PAS-based colorimetric assay. Combined data from two independent experiments are shown as means ± SEM. (**d**) Heatmap showing scaled relative expression levels of polysaccharide utilization loci (PULs) in *B. theta* RNA transcripts sequenced from stool collected from *B. theta* (ATCC 29148)-colonized gnotobiotic mice with or without co-administration of *B. ovatus*. Transcripts were evaluated on day 14 after bacterial introduction to germ-free mice. Right: Significantly altered PULs and their substrates.

Using network analysis, we investigated for potential interactions between gene expression of *B. ovatus* and *B. theta*. Given our hypothesis that *B. ovatus* was performing metabolic functions that inhibited *B. theta* utilization of mucins, we were particularly interested in PULs of *B. ovatus* that were negatively associated with PULs of *B. theta* that were related to the degradation of mucin O-glycans such as PULs 12, 14, 16, 72, 78, and 81 (Fig. 6a and Extended Data Fig. 5b). Interestingly, one of the PULs of *B. theta* that participates in the degradation of mucin O-glycans, PUL14, was negatively correlated with multiple PULs of *B. ovatus* including PUL73, −100, and 108. Another PUL of *B. theta* that participates in the degradation of mucin O-glycans, PUL78, was also negatively correlated with multiple PULs of *B. ovatus* including PUL42, −47, −73, −100, and −108. Both PUL47 and PUL73 of *B. ovatus* have been reported to contribute the degradation of xylose-comprising polysaccharides including xyloglucan, wheat arabinoxylan, oat spelt xylan, and complex xylans^26,27^. This led us to ask if degradation of xylose-comprising polysaccharides by *B. ovatus* could suppress mucin glycan utilization by *B. theta* in minimal media supplemented with porcine gastric mucin. We evaluated the effects of combining this media with media conditioned by *B. ovatus* for 48 hours in the presence of various polysaccharides (Fig. 6b). Interestingly, *B. ovatus* culture media containing wheat arabinoxylan or tamarind xyloglucan each significantly suppressed the growth as well as mucin degradation by *B. theta*, while *B. ovatus* culture medium supplemented with starch, which is not composed of xylose, did not suppress the growth and mucin degradation by *B. theta* (Fig. 6c). Finally, we asked what effects *B. ovatus* had on the gene expression of *B. theta*. To evaluate this, we turned to gnotobiotic mice and evaluated fecal RNA transcripts in germ-free mice 2 weeks after introducing either *B. theta* alone or *B. ovatus* as well as *B. theta*. We found that introduction of *B. ovatus* resulted in *B. theta* significantly downregulating PULs 12 and 73, both of which contribute to degradation of mucin O-glycans (Fig. 6d). Altogether, these data suggested that introduction of *B. ovatus* into meropenem-treated allo-HSCT mice resulted in a carbohydrate-enriched intestinal environment in the colonic lumen by degrading dietary-derived polysaccharides such as xylose-comprising polysaccharides, leading to inhibition of *B. theta* mucin utilization, ultimately resulting in amelioration of disrupted microbiota-induced severe GVHD.

## Discussion

Allo-HSCT is a curative therapy for high-risk hematological malignancies, but complications such as infections and GVHD continue to limit its success. The intestinal microbiota is an important modulator of GVHD, and broad-spectrum antibiotics are known to increase the incidence of aGI-GVHD by compromising several functions of an intact intestinal microbiota, resulting in alterations to the intestinal environment including reduced concentrations of metabolic products in the colonic lumen^15^. The poor prognosis of severe aGI-GVHD underlines the need to better understand how intestinal microbes can help suppress GVHD in allo-HSCT.

In this study, we investigated the impact of the intestinal microbiota on treatment responsiveness of aGI-GVHD using clinical microbiome data. In our retrospective analysis of the fecal microbiome in aGI-GVHD patients, we found that an altered microbiome profile at presentation of aGI-GVHD and a history of treatment with carbapenem-class antibiotics such as meropenem were significantly associated with developing steroid-refractory GVHD, whereas a high abundance of the commensal species *B. ovatus*, commonly found in normal individuals, was significantly associated with improved GVHD response to steroid therapy. Consistent with this result, *B. ovatus* has previously been associated with reduced incidence of GVHD^28^. However, it has not been well studied whether *B. ovatus* can mechanistically suppress severe GVHD.

Some prior studies have reported that *B. ovatus* can mediate multiple beneficial functions in maintaining intestinal homeostasis in the host via production of indole-3-acetic acid or sphingolipid production^25,29^. Here, in a murine model, we found that introduction of *B. ovatus* resulted in improved survival in meropenem-treated allo-HSCT mice but not in meropenem-untreated allo-HSCT mice. This suggested that *B. ovatus* helped suppress GVHD only in hosts with a disrupted microbiota, and that a key function of *B. ovatus* may be related to mechanisms underlying aggravated colonic GVHD in the setting of antibiotic injury. Unlike *B. ovatus*, *B. theta* is known to be capable of utilizing host-derived glycans^30,31^, and was found to aggravate colonic GVHD in our prior study^15^. In this study, we found that in the setting of an antibiotic-disrupted microbiota with expansion of mucus-degrading *B. theta*, the introduction of *B. ovatus* ameliorated the severity of colonic GVHD via polysaccharide degradation, thus producing abundant monosaccharides and improving the intestinal metabolomic environment in allo-HSCT.

As limitations, this clinical microbiome analyses were retrospectively performed with relatively small numbers in our cohort. The timing of stool collection relative to allo-HSCT was different in each patient, so the effects of antibiotic exposure during allo-HSCT and the impacts of microbiota disruption due to antibiotics were potentially different individually. Also, we found that steroid-refractory patients showed significantly higher histological GVHD grades of the colon than steroid-responsive patients did. This could mean that severe mucosal injury from GVHD may in itself cause a dysbiotic microbiota and also be associated with higher likelihood of steroid-resistance.

In order to better determine causality, we conducted a murine GVHD model combined with in vitro assays and were able to confirm that *B. ovatus* ameliorated meropenem-aggravated colonic GVHD via xylose-comprising polysaccharide degradation. However, *B. ovatus* has a broad ability to evoke not only carbohydrate degradation but also production of tryptophan metabolites^25^, sphingolipids^29^, and bile salt hydrolase^32^ and secretion of fecal immunoglobulin A^33^. In addition, although we confirmed that *B. ovatus* could ameliorate GVHD caused by dysbiotic microbiota in a murine model, we are still not sure whether *B. ovatus* is also associated with the efficacy of steroid therapy for aGI-GVHD. Further studies will be needed to fully understand the influence the intestinal microbiota plays with regard to response to therapy.

In summary, an antibiotic-disrupted microbiota caused by carbapenems including meropenem increased the severity of intestinal GVHD and was associated with treatment-refractory aGI-GVHD in patients. Mouse modeling demonstrated that introducing *B. ovatus* can ameliorate the severity of GVHD in a model of meropenem-aggravated colonic GVHD. This understanding of how specific bacteria such as *B. ovatus* can reduce intestinal inflammation should facilitate the development of new strategies to better prevent and treat this important limitation of allo-HSCT.

## Methods

### Retrospective study design

A total of 37 aGI-GVHD patients who underwent allo-HSCT during 2017 to 2019 at MD Anderson Cancer Center provided stool samples for our biorepository, and these patient stool samples were analyzed retrospectively. Acute GVHD was diagnosed by clinical and/or pathological findings and graded according to standard criteria^34^. These patients included 28 with classic aGI-GVHD and 9 with late-onset aGI-GVHD by National Institutes of Health consensus criteria^17^. We classified patients by steroid responsiveness to GVHD, including 20 patients who were steroid-responsive and 17 patients who were steroid-refractory. We determined treatment response as previously reported^18^: briefly, a lack of response on the basis of organ assessment after at least 3 days of high-dose systemic glucocorticoid therapy; a lack of improvement after 7 days; or treatment failure during steroid tapering or an inability to taper the dose to <0.5 mg/kg/day of methylprednisolone. All patients received initial therapy with methylprednisolone or prednisone at 2 mg/kg/day followed by tapering per institutional guidelines. Signed informed consent was provided by all study participants including healthy volunteers, and this study was approved by The University of Texas MD Anderson’s Institutional Review Board.

### Human samples

Samples were collected from patients undergoing allo-HSCT and healthy volunteers and stored at 4°C for 24–48 hours until aliquoted for long-term storage at −80°C.

### Mice

Female C57BL/6J (B6: H-2^b^) and B6D2F1 (H-2^b/d^, CD45.2^+^) were purchased from The Jackson Laboratory (Bar Harbor, ME). Eight- to 12-week-old female C57BL/6 germ-free mice for murine studies were provided by the gnotobiotic facility of Baylor College of Medicine (Houston, TX). All animal experiments were performed under the Guide for the Care and Use of Laboratory Animals Published by the National Institutes of Health and was approved by the Institutional Animal Care and Use Committee. Experiments in this manuscript were performed in a non-blinded fashion.

### Antibiotics administration

Meropenem was dissolved with phosphate buffer, pH 8.0, and given at a concentration of 0.625 g/L in drinking water from day 3 to day 15 after transplant.

### HSCT

Mice received transplants as previously described^35^. In brief, after receiving myeloablative total-body irradiation (11 Gray) delivered in 2 doses at 4-hour intervals, B6D2F1 (H-2^b/d^) mice were intravenously injected with 5 × 10^6^ bone marrow cells and 5 × 10^6^ splenocytes from allogeneic B6 (H-2^b^) donors. Female mice that were 8 to 12 weeks old were allocated randomly to each experimental group, ensuring the mean body weight in each group was similar. Total body radiotherapy was performed using a Shepherd Mark I, Model 30, ^137^Cs irradiator. Mice were maintained in specific pathogen-free (SPF) conditions and received normal chow (LabDiet PicoLab Rodent Diet 20 5053, Lab Supply). Survival after HSCT was monitored daily, and the degree of clinical GVHD was assessed weekly using an established scoring system^36^.

### Histological and immunohistochemistry analysis

For evaluation of mucus thickness, colonic sections containing stool pellets were fixed in methanol-Carnoy fixative composed of methanol (60%), chloroform (30%) and glacial acetic acid (10%) and 5 μm sections were made and stained with periodic acid-Schiff (PAS). Sections were imaged using an Aperio AT2. Mucus thickness of the colonic sections was measured using eSlide Manager Version 12.4.3.5008. Eight measurements per image were taken and averaged over the entire usable colon surface.

### Sequencing of 16S rRNA gene amplicons

Fecal samples that were collected from patients and mice were weighed before DNA isolation. In brief, genomic DNA was isolated using the QIAamp DNA mini kit (51306, Qiagen) according to the manufacturer’s protocol, which was modified to include an intensive bead-beating lysis step. The V4 region of the 16S rRNA gene was amplified by PCR from 100 ng of extracted genomic DNA using 515 forward and 806 reverse primer pairs^37^. The quality and quantity of the barcoded amplicons were assessed on an Agilent 4200 TapeStation system and Qubit Fluorometer (Thermo Fisher Scientific), and libraries were prepared after pooling at equimolar ratios. The final libraries were purified using QIAquick gel extraction kit (28706X4, Qiagen) and sequenced with a 2 × 250 base pair paired-end protocol on the Illumina MiSeq platform.

### Microbiome data analysis

Sequencing data from paired-end reads were de-multiplexed using QIIME 2^38^. Merging of paired-end reads, dereplicating, and length filtering was performed using VSEARCH 2.17.1^39^. Following de-noising and chimera calling using the unoise3 command^40^, unique sequences were taxonomically classified with mothur^41^ using the Silva database^42^ version 138. Weighted UniFrac distances^43^ were determined using QIIME 2, visualized using PCoA, and evaluated for statistical significance using PERMANOVA testing. For differential abundance analysis, abundances of sequences belonging to taxonomical groups were included for analysis using DESeq2 and adjusted for multiple comparisons using the method of Benjamini and Hochberg. Patient microbiome data were classified into 2 clusters using the hcluster function by the amap library of R.

### Quantification of fecal bacterial density

Genomic DNA was isolated from stool as described above. qPCR was performed as previously described^44^. In brief, 16S rRNA gene sequences were amplified from total fecal DNA using the primers 926F (5′-AAACTCAAAKGAATTGACGG-3′) and 1062R (5′-CTCACRRCACGAGCTGAC-3′). Real-time PCR was carried out in 96-well optical plates on QuantStudio Flex 6 RT-PCR (Thermo Fisher) and KAPA SYBR FAST Master Mix (Roche). The PCR conditions included one initial denaturing step of 10 min at 95°C and 40 cycles of 95°C for 20 sec and 60°C for 1 min. Melting-curve analysis was performed after amplification. To determine bacterial density, a plasmid with a 16S rRNA gene of a murine *Blautia* isolate was generated in the pCR4 backbone and used as a standard.

### Culturing of bacteria

*Bacteroides ovatus* (MDA-HVS BO001) was isolated and cultured from healthy volunteer’s stool samples in a Whitley anaerobic chamber (10% H_2_, 5% CO_2_ and 85% N_2_). Human-derived *B. ovatus* (ATCC 8483) and human-derived *B. theta* (ATCC 29148) were purchased from American Type Culture Collection (ATCC). Mouse-derived BT (MDA-JAX BT001) was previously isolated^15^. Bacterial number was quantified using a Nexcelom Cellometer cell counter with SYTO BC dye and propidium iodide. Bacterial growth experiments were performed in a liquid media, BYEM10, composed of a hybrid of BHI and M10 supplemented with yeast extract as previously described^15,45^. Bacteria were cultured up to 24 or 48 hours at a starting concentration of 1 × 10^6^ bacteria/ml in BYEM10 broth (pH 7.2) with or without 5 mg/ml of porcine gastric mucin (M1778, Sigma-Aldrich), wheat arabionoxylan (wheat flour; low viscosity; Megazyme), xylan (Beechwood; Megazyme), xyloglucan (Tamarind; Megazyme), or starch (wheat; Sigma-Aldrich). Optical densities (OD_600 nm_) of bacterial cultures were measured with a BioTek Epoch 2 plate reader.

### Mucin degradation assay

Levels of mucin glycans in culture supernatants were determined by a PAS-based colorimetric assay as previously described^15,45^. Briefly, culture supernatants were centrifuged at 20,000*g* for 10 minutes at 4°C and collected. To perform mucin precipitation, 500 μl of culture supernatants was mixed with 1 ml of molecular grade ethanol and incubated at −30°C for overnight. Culture supernatants were centrifuged at 20,000*g* for 10 minutes at 4°C. Mucin-containing pellets were washed with 1 ml of molecular grade ethanol twice and resuspended in 500 μl of PBS. A total of 10 μl of washed culture supernatants was transferred into a round-bottom 96-well plate containing 15 μl of PBS. Serially diluted porcine gastric mucin (Sigma-Aldrich) standards were prepared. Freshly prepared 0.06% periodic acid in 7% acetic acid was added and incubated at 37°C for 90 min, followed by 100 μl of Schiff’s reagent (84655, Sigma-Aldrich) and incubation at room temperature for 40 min. Absorbance was measured at 550 nm using a BioTek Synergy HTX plate reader.

### Analysis of carbohydrates by IC-MS

To determine the relative abundance of carbohydrates in mouse fecal samples, extracts were prepared and analyzed by ultrahigh-resolution mass spectrometry. Fecal pellets were homogenized with a Precellys Tissue Homogenizer. Metabolites were extracted using 1 ml of ice-cold 80/20 (v/v) methanol/water. Extracts were centrifuged at 17,000*g* for 5 min at 4°C, and supernatants were transferred to clean tubes, followed by evaporation to dryness under nitrogen. Dried extracts were reconstituted in deionized water, and 5 μl was injected for analysis by IC-MS. IC mobile phase A (MPA; weak) was water, and mobile phase B (MPB; strong) was water containing 100 mM KOH. A Thermo Scientific Dionex ICS-5000+ system included a Thermo CarboPac PA20-Fast column (4 μm particle size, 100 × 2 mm) with the column compartment kept at 30°C. The autosampler tray was chilled to 4°C. The mobile phase flow rate was 200 μl/min, and the gradient elution program was: 0–0.5 min, 1% MPB; 0.5–10 min, 1%−5% MPB; 10–15 min, 5%−95% MPB; 15–20 min, 95% MPB; 20.5–25, 95–1% MPB. The total run time was 25 min. To assist the desolvation for better sensitivity, methanol was delivered by an external pump and combined with the eluent via a low dead volume mixing tee. Data were acquired using a Thermo Orbitrap Fusion Tribrid Mass Spectrometer under ESI negative ionization mode at a resolution of 240,000. Raw data files were imported to Thermo TraceFinder and Compound Discoverer software for spectrum database analysis. The relative abundance of each metabolite was normalized by sample weight.

### Analysis of tryptophan metabolites by LC-HRMS

To determine the relative concentration of tryptophan metabolites in mouse fecal samples, extracts were prepared and analyzed by liquid chromatography coupled with high-resolution mass spectrometry (LC-HRMS). Approximately 50 mg of stool was pulverized on liquid nitrogen, then homogenized with Precellys Tissue Homogenizer. Metabolites were extracted using 0.5 ml of ice-cold 50/50 (v/v) methanol/acetonitrile followed by 0.5 mL 0.1% formic acid in 50/50 (v/v) Acetonitrile/Water. Extracts were centrifuged at 17,000*g* for 5 min at 4°C, and supernatants were transferred to clean tubes, followed by evaporation to dryness under nitrogen. Samples were then reconstituted in 50/50 (v/v) methanol/water, then 10 μl was injected into a Thermo Vanquish liquid chromatography (LC) system containing a Waters XSelect HSS T3 2.1 × 150 mm column with 2.5-μm particle size. MPA was 0.1% formic acid in water. MPB was 100% methanol. The flow rate was 200 μl/min (at 35°C), and the gradient conditions were: initial 5% MPB, increased to 95% MPB at 15 min, held at 95% MPB for 5 min, and returned to initial conditions and equilibrated for 5 min. The total run time was 25 min. Data were acquired using a Thermo Orbitrap Fusion Tribrid mass spectrometer under ESI positive and negative ionization modes at a resolution of 240,000 with full scan mode. Raw data files were imported into Thermo TraceFinder software for final analysis. The relative concentration of each compound was normalized by stool weight.

### Whole-genome sequencing of patient fecal samples

Genomic DNA was isolated from patient fecal samples and purified using a Qiagen Genomic-tip 20/G column, according to the manufacturer’s instructions. For short-read Illumina sequencing, libraries were constructed with a Nextera DNA Flex Library Prep Kit (Illumina), according to the manufacturer’s protocol. All libraries were quantified with a TapeStation and pooled in equal molar ratios. The final libraries were sequenced with the NovaSeq 6000 platform (Illumina) to produce 2×150 bp paired-end reads, resulting in ~5 Gb per sample. In sequencing analysis, sequence reads were filtered by their quality using the VSEARCH 2.17.1. The abundance of taxa, microbial metabolic pathways, and gene expression was profiled by the HUMAnN3. Differential expression profiles were analyzed by the DESeq2 package in R.

### Whole-genome sequencing of *B. ovatus* (MDA-HVS BO001)

*B. ovatus* (MDA-HVS BO001) genomic DNA was isolated and purified using a Qiagen Genomic-tip 20/G column, according to the manufacturer’s instructions. For short-read Illumina sequencing, libraries were constructed with a Nextera DNA Flex Library Prep Kit (Illumina, San Diego, CA, USA), according to the manufacturer’s protocol. All libraries were quantified with a TapeStation and pooled in equal molar ratios. The final libraries were sequenced with the NovaSeq 6000 platform (Illumina) to produce 2×150 bp paired-end reads, resulting in ~5 Gb per sample. For long-read Nanopore sequencing, 500 ng of genomic DNA was used for library preparation using the Rapid Sequencing Kit (SQK-RAD004, Oxford Nanopore Technologies). Libraries were loaded into a FLO-MIN106 flow-cell for a 24-h sequencing run on a MinION sequencer platform (Oxford Nanopore Technologies, Oxford, UK). Data acquisition and real-time base calling were carried out by the MinKNOW software version 3.6.5. The fastq files were generated from basecalled sequencing fast5 reads.

### Hybrid assembly and genome annotation of *B. ovatus* (MDA-HVS BO001)

To assemble the complete genome of *B. ovatus*, Flye version 2.8.2^46^ was used with long reads (Nanopore) and short reads (NovaSeq) combined using default settings. The similarities of the genome of MDA-HVS BO001 to other reference genomes was calculated using blastn for *B*. (ATCC 8483)^47^. Open reading frames of *B. ovatus* (MDA-HVS BO001) were identified using prokka^48^. The genome of *B. ovatus* and open reading frames were depicted using DNA plotter software^49^.

### RNA sequencing and analysis

Approximately 30 mg of stool was freshly collected in 700 μl of ice-cold QIAzol containing 200 μl of 0.1-mm-diameter Zirconia Silica beads (11079101z, BioSpec). Samples were bead beaten twice for 2 min with a 30-s interval recovery. Samples were then centrifuged at 12,000*g* for 1 min, and the supernatant was collected for RNA isolation using the RNeasy mini kit (74104, Qiagen). RNA was treated on column with DNase I (79254, Qiagen) to eliminate contaminating genomic DNA. RNA quantity and quality were determined using an Agilent 4200 TapeStation system (Agilent). A total of 250 ng of total RNA from mouse stools was used to construct libraries using the Universal Prokaryotic RNA-Seq Library Preparation Kit (9367–32, Tecan) with Unique Dual Indexes (S02480-FG, Tecan), following the manufacturer’s protocol. The cDNA libraries were sequenced on the Illumina NovaSeq 6000 system to produce 2 × 150 bp paired-end reads. Sequence data were demultiplexed using QIIME 2^38^ and their qualities were checked using VSEARCH 2.17.1^39^. Data were filtered and truncated by quality with VSEARCH default settings. The total reads of mouse stool samples were 160896223 ± 93489752 (mean ± standard deviation). Sequences of ribosomal RNA were removed using BWA software against prokaryotic ribosomal RNA sequences from prokaryotic RefSeq genomes^50^. Sequences of interest were further identified using diamond software version 0.9.24^51^ to align against PULs. Features with percentage identity less than 80% were excluded. The total counts of bacterial isolated samples were 360932 ± 284308 and 966485 ± 617495 in *B. ovatus* and *B. theta*, respectively (mean ± standard deviation). Aligned mRNA expression changes were calculated using the DESeq2 in R software version 4.1.2 via RStudio version 2022.02.0 Build 443. P values < 0.05 were considered statistically significant.

### Network analysis using bacterial RNA transcripts

The expressions of PULs of *B. theta* and *B. ovatus* in meropenem-untreated and -treated mice that received *B. ovatus* were standardized by relative abundances in each sample. PULs with average expression rates of 1% or less in each group were excluded from the network analysis. Each data set was logit transformed, and then r and *p* values were calculated by Pearson correlation analysis between *B. theta* and *B. ovatus* PULs. *P* values were corrected by false discovery rate (FDR). PUL combinations showing a corrected *p*-value of 0.05 or less with a negative r value were depicted using Cytoscape^52^ for *B. theta* PULs known to degrade mucin-O-glycan.

### Statistical analysis

Data were checked for normality and similar variances between groups, and Student t-tests were used when appropriate. Mann-Whitney U tests were used to compare data between two groups when the data did not follow a normal distribution. Kaplan-Meier curves were used to depict survival probabilities, and the log-rank test was applied to compare survival curves. For clinical data analysis, non-repeated ANOVA was used to compare continuous variables, while chi-square or Fisher exact tests were used to analyze the frequency distribution between categorical variables. Analyses were performed using R software version 4.1.2 and Prism version 9.0 (GraphPad Software). P values < 0.05 were considered statistically significant.

## Extended Data

## Supplementary Material

1
