# Supplementary material for: Bacteroides ovatus alleviates dysbiotic microbiota-induced intestinal graft-versus-host disease
Source: Res Sq. 2023 Jan 31:rs.3.rs-2460097. Preprint. [Version 1] doi: 10.21203/rs.3.rs-2460097/v1 (PMC9915792; doi:10.21203/rs.3.rs-2460097/v1)
Supplement: 1 [file NIHPPRS2460097V1-supplement-1.pdf]

Supplemental information

Table S1. Patient characteristics of all allo-HSCT patients with aGI-GVHD.

	n = 37
Median age (range), y	55 (22-74)
Male, n (%)	26 (70%)
Donor type, n (%)	
MRD	11 (30%)
MUD	20 (54%)
Haplo	6 (16%)
Cell source, n (%)	
Bone marrow	4 (11%)
Peripheral blood	33 (89%)
Conditioning, n (%)	
Myeloablative	24 (65%)
Non-myeloablative	13 (35%)
GVHD prophylaxis, n (%)	
PTCy/Tacrolimus	8 (22%)
PTCy/Tacrolimus/MMF	8 (22%)
Tacrolimus/MTX	9 (24%)
Tacrolimus/MTX/ATG	5 (14%)
Tacrolimus/MMF	5 (14%)
Tacrolimus/MMF/ATG	2 (5%)
Median day of aGI-GVHD onset (range)	36 (13-367)
aGI-GVHD clinical stages, n (%)	
Stage 0-2	25 (68%)
Stage 3-4	11 (30%)
Unknown	1 (3%)
Histology grades of the colon, n (%)	
Grade 0-2	28 (76%)
Grade 3-4	9 (24%)

ATG, anti-thymocyte globulin; GVHD, graft-versus-host disease; aGI-GVHD, acute gastrointestinal GVHD; Haplo, human leukocyte antigen (HLA)-haploidentical related donor; MRD, HLA-matched related donor; MTX, methotrexate; MMF, mycophenolate mofetil; MUD, HLA-matched unrelated donor; PTCy, post-transplant cyclophosphamide.

Table S2. Patient characteristics of allo-HSCT patients who were classified into clusters 1 and 2 by intestinal microbiome profiling at the onset of aGI-GVHD.

	Cluster 1 (n = 9)	Cluster 2 (n = 28)	*P* value
Median age (range), y	60 (46-69)	51 (22-74)	0.05
Male, n (%)	7 (78%)	19 (66%)	0.01
Donor type, n (%)			0.9
MRD	3 (33%)	8 (28%)	
MUD	5 (56%)	15 (54%)	
Haplo	1 (11%)	5 (18%)	
Cell source, n (%)			1.0
Bone marrow	1 (11%)	3 (11%)	
Peripheral blood	8 (89%)	25 (89%)	
Conditioning, n (%)			1.0
Myeloablative	6 (66%)	18 (64%)	
Non-myeloablative	3 (33%)	10 (36%)	
GVHD prophylaxis, n (%)			0.9
PTCy/Tacrolimus	2 (22%)	6 (21%)	
PTCy/Tacrolimus/MMF	3 (33%)	5 (18%)	
Tacrolimus/MTX	3 (33%)	6 (21%)	
Tacrolimus/MTX/ATG	0 (0%)	5 (18%)	
Tacrolimus/MMF	0 (0%)	5 (18%)	
Tacrolimus/MMF/ATG	1 (11%)	1 (3%)	
Median day of aGI-GVHD onset (range)	27 (14-127)	41 (13-367)	0.04
aGI-GVHD clinical stages, n (%)			0.8
Stage 0-2	7 (77%)	18 (64%)	
Stage 3-4	2 (22%)	9 (32%)	
Unknown	0 (0%)	1 (4%)	
Histology grades of the colon, n (%)			0.4
Grade 0-2	8 (88%)	20 (71%)	
Grade 3-4	1 (11%)	8 (29%)	

Non-repeated ANOVA was used to compare continuous variables, while chi-square or Fisher exact test was used to analyze the frequency distribution between categorical variables. *P*-value under 0.05 was considered statistically significant. ATG, anti-thymocyte globulin; GVHD, graft-versus-host disease; aGI-GVHD, acute gastrointestinal GVHD; Haplo, human leukocyte antigen (HLA)-haploidentical related donor; MRD, HLA-matched related donor; MTX, methotrexate; MMF, mycophenolate mofetil; MUD, HLA-matched unrelated donor; PTCy, post-transplant cyclophosphamide.

Table S3. Patient characteristics of allo-HSCT patients who underwent intestinal microbiome profiling at the onset of aGI-GVHD.

	Steroid-responsive (n = 20)	Steroid-refractory (n = 17)	*P* value
Median age (range), y	62 (46-74)	45 (22-71)	0.0001
Male, n (%)	11 (55%)	13 (77%)	0.3
Donor type, n (%)			0.6
MRD	5 (25%)	6 (35%)	
MUD	13 (65%)	7 (41%)	
Haplo	2 (10%)	4 (24%)	
Cell source, n (%)			1.0
Bone marrow	2 (10%)	2 (10%)	
Peripheral blood	18 (86%)	17 (85%)	
Conditioning, n (%)			0.7
Myeloablative	12 (60%)	12 (71%)	
Non-myeloablative	8 (40%)	5 (29%)	
GVHD prophylaxis, n (%)			0.5
PTCy/Tacrolimus	7 (35%)	1 (6%)	
PTCy/Tacrolimus/MMF	4 (20%)	4 (24%)	
Tacrolimus/MTX	4 (20%)	5 (29%)	
Tacrolimus/MTX/ATG	1 (5%)	4 (24%)	
Tacrolimus/MMF	2 (10%)	3 (18%)	
Tacrolimus/MMF/ATG	2 (10%)	0 (0%)	
Median day of aGI-GVHD onset (range)	32 (14-367)	42 (13-253)	0.13
aGI-GVHD clinical stages, n (%)			0.03
Stage 1-2	18 (90%)	7 (41%)	
Stage 3-4	2 (10%)	9 (53%)	
Unknown	0 (0%)	1 (5%)	
Histology grades of the colon, n (%)			0.005
Grade 0-2	19 (95%)	9 (53%)	
Grade 3-4	1 (5%)	8 (47%)	

Non-repeated ANOVA was used to compare continuous variables, while chi-square or Fisher exact test was used to analyze the frequency distribution between categorical variables. *P*-value under 0.05 was considered statistically significant. ATG, anti-thymocyte globulin; GVHD, graft-versus-host disease; aGI-GVHD, acute gastrointestinal GVHD; Haplo, human leukocyte antigen (HLA)-haploidentical related donor; MRD, HLA-matched related donor; MTX, methotrexate; MMF, mycophenolate mofetil; MUD, HLA-matched unrelated donor; PTCy, post-transplant cyclophosphamide.
